# A point mutation in AgrC determines cytotoxic or colonizing properties associated with phenotypic variants of ST22 MRSA strains

**DOI:** 10.1038/srep31360

**Published:** 2016-08-11

**Authors:** Srikanth Mairpady Shambat, Nikolai Siemens, Ian R. Monk, Disha B. Mohan, Santhosh Mukundan, Karthickeyan Chella Krishnan, Sushma Prabhakara, Johanna Snäll, Angela Kearns, Francois Vandenesch, Mattias Svensson, Malak Kotb, Balasubramanian Gopal, Gayathri Arakere, Anna Norrby-Teglund

**Affiliations:** 1 Karolinska Institutet, Center for Infectious Medicine, Karolinska University Hospital Huddinge, 141 86, Sweden; 2 Department of Microbiology and Immunology, University of Melbourne, Victoria, 3010, Australia; 3 Molecular Biophysics Unit, Indian Institute of Science, Bangalore, 560012, India; 4 Department of Basic Sciences, School of Medicine and Health Sciences, University of North, Dakota, ND, 58202-9037, USA; 5 Society for Innovation and Development, Indian Institute of Science, Bangalore, 560012, India; 6 National Infection Service, Public Health England (PHE), Colindale, London, NW95EQ, UK; 7 CIRI, International Center for Infectiology Research, Inserm, U1111, CNRS UMR5308, Université Lyon 1, École Normale, Supérieure de Lyon, French; 8 French National Reference Center for Staphylococci, Hospices Civils de Lyon, 69677, Bron Cedex, France

## Abstract

Methicillin-resistant *Staphylococcus aureus* (MRSA) is a major cause of skin and soft tissue infections. One of the highly successful and rapidly disseminating clones is MRSA ST22 commonly associated with skin tropism. Here we show that a naturally occurring single amino acid substitution (tyrosine to cysteine) at position 223 of AgrC determines starkly different ST22 *S. aureus* virulence phenotypes, e.g. cytotoxic or colonizing, as evident in both *in vitro* and *in vivo* skin infections. Y223C amino acid substitution destabilizes AgrC-AgrA interaction leading to a colonizing phenotype characterized by upregulation of bacterial surface proteins. The colonizing phenotype strains cause less severe skin tissue damage, show decreased susceptibility towards the antimicrobial LL-37 and induce autophagy. In contrast, cytotoxic strains with tyrosine at position 223 of AgrC cause infections characterized by inflammasome activation and severe skin tissue pathology. Taken together, the study demonstrates how a single amino acid substitution in the histidine kinase receptor AgrC of ST22 strains determines virulence properties and infection outcome.

*Staphylococcus aureus* has long been recognized as a major human pathogen causing both hospital-associated and community-acquired (CA) infections. The health burden associated with these infections is further heightened by the emergence of methicillin-resistant *S. aureus* (MRSA) worldwide^1^,^2^,^3^,^4^. Epidemiologic studies from the US have identified MRSA as the major cause of skin and soft tissue infections^5^,^6^. In addition, Klevens *et al*.^2^ reported that MRSA causes more deaths in the US than any other infectious disease including HIV/AIDS.

The spread of MRSA worldwide is due to the dissemination of a limited number of MRSA clones (reviewed in^7^). Epidemic MRSA-15 (EMRSA-15), belonging to the multilocus sequence type (ST) ST22 is recognized as a particularly successful, rapidly disseminating clone that has spread across continents^8^,^9^. In the UK, EMRSA-15 emerged in the early 1990s and 10 years later, it accounted for 60% of all MRSA nosocomial bacteremia cases^10^. In a recent study comparing *S. aureus* bloodstream infections from 2011 with 2006 data, ST22 was identified as the most markedly expanding MRSA clone in Europe^11^. Taken together with reports of Panton-Valentine Leukocidin (PVL)-positive ST22 strains associated with outbreaks of neonatal infections^12^ as well as skin and soft tissue infections^13^,^14^,^15^, the rapid expansion of these ST22 strains is a concerning development.

*S. aureus* expresses a wide array of virulence factors, including among others secreted toxins that are strongly implicated in the pathogenic mechanisms underlying toxic shock, severe pneumonia and complicated skin and soft tissue infections^16^,^17^. These include superantigens (staphylococcal enterotoxins and TSST-1), and cytotoxins such as PVL, α-toxin, and the phenol soluble modulins (PSM)^16^,^17^,^18^. Apart from that *S. aureus* can also express a broad range of cell surface virulence factors that are covalently attached to peptidoglycan, known as cell wall-anchored proteins^19^. These surface proteins play a major role in promoting the bacterial adhesion and invasion of extracellular matrix and host cells, and are critical in determining the success of the bacterium as a colonizer, persister and/or pathogen^19^,^20^. A key regulator of virulence factor expression is the accessory gene regulator (*agr)* quorum-sensing system composed of AgrA (response regulator), B (integral membrane protease), C (histidine kinase), and D (auto-inducing peptide)^21^. Clinical isolates exhibiting *agr*-defective phenotypes have been reported, with dysfunction being beneficial for survival and persistence within the infected host but counter selected for against transmission^22^,^23^,^24^,^25^. Also, recent studies have emphasized the impact of differential expression of the toxins on the strains’ virulence properties^26^,^27^.

In our previous study, we identified two ST22 MRSA strains with the same genetic background (ST22, agrI, PVL+, *mec*A+, identical virulence gene profile) that displayed starkly different phenotypic response profiles, i.e. either proliferative or cytotoxic responses^26^. In this report, whole genome sequencing was applied to these clinical ST22 variants. The analyses identified a specific mutation in *agrC,* which contributed to differential virulence profiles in *in vitro* cell and tissue model systems, as well as *in vivo* in a murine tissue infection model. We show that this specific AgrC variation determines cytotoxic or colonizing infection profiles in infected tissue.

## Results

### Distinct phenotypic virulence profiles of ST22 strains associated with a naturally occurring single point mutation in *agrC*

In the report by Mairpady Shambat *et al*.^26^, different phenotypic response profiles were noted in peripheral blood mononuclear cells (PBMC) exposed to varying *S. aureus* strains from different genetic backgrounds and even within the same ST type such as ST22 (Supplementary Table 1). Here we focused on two ST22 MRSA skin isolates, specifically: M37 isolated from recurrent skin infection and PUNE08 isolated from an acute soft tissue infection. The strains had identical virulence gene profiles as determined by DNA microarray^28^ (Supplementary Table 2), and yet displayed starkly different phenotype profiles (cytotoxic versus proliferative).

**Figure 1:**
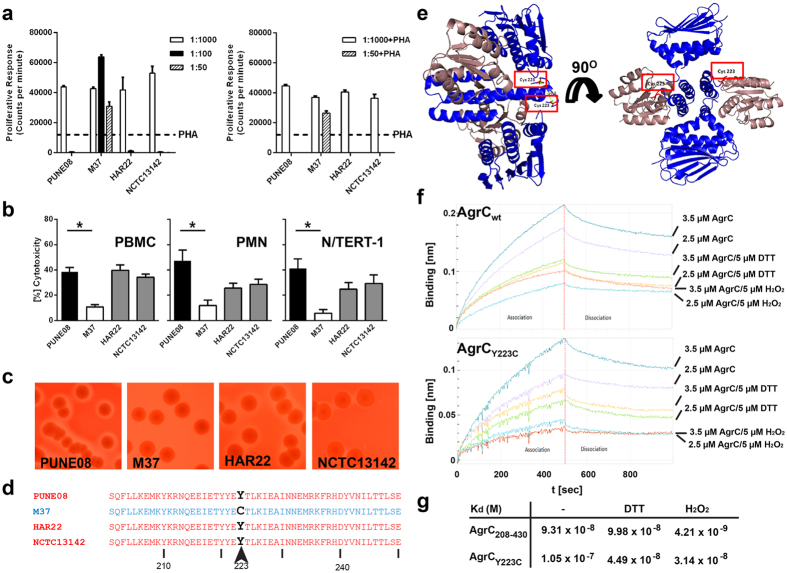
Natural amino acid sequence variation in AgrC results in different phenotypic profiles of clinical *S. aureus* strains. (**a**) Proliferative or cytotoxic responses by human peripheral blood mononuclear cells (PBMC) isolated from three healthy donors stimulated with indicated dilution series of bacterial supernatants prepared from overnight cultures of *S. aureus* strains. Stimulation without (left panel) and with (right panel) addition of PHA to each supernatant are shown. The data represent the mean values ± s.d. (n  =  3). (**b**) Cytotoxicity induced by bacterial supernatants of 1:50 dilutions towards PBMC, human neutrophils (PMN) and human keratinocytes (N/TERT-1). Mean values ± s.d. from four or more volunteers (PBMC, PMN) or experiments are shown (n ≥ 4). The statistical significance between PUNE08 and M37 strains was determined using two-tailed Mann-Whitney U-test (*p < 0.05). (**c**) Representative images of hemolysis on blood agar plates induced by indicated clinical *S. aureus* strains (see also Supplementary Fig. 1e). (**d**) Amino acid sequence analysis of AgrC from indicated strains at specified positions. (**e**) Theoretical side and front representation of AgrC_Y223C_ protein dimeric structure showing two cysteines at 223^rd^ position which are placed ~15 Å apart. (**f**) Quantification of the interaction of recombinant wild-type AgrC (upper panel) and AgrC_Y223C_ (lower panel) (analytes) with immobilized AgrA (ligand) using bio-layer interferometry. (Note the difference in scale between upper panel and lower panel). Representative profiles of the relative bio-layer Interferometry responses for the association and dissociation of different analyte concentrations in combination with DTT and H_2_O_2_ are shown (n = 2). (**g**) The values for dissociation constant (*K*_*d*_) were calculated from the binding data with the Octet Data Analysis Software v 8.0.

First, we compared their phenotypic profile with two EMRSA-15 reference strains, HAR22 and NCTC13142. PBMC from three donors were stimulated with serial dilutions of bacterial overnight culture supernatants and proliferative activity was determined. Supernatant from strain M37 induced proliferation at all dilutions tested, whereas PUNE08 and the reference strains induced proliferation only at the highest dilution (1:1000) (Fig. 1a). To test whether the lack of proliferation in more concentrated supernatants was due to cytotoxicity, proliferation was assessed following stimulations of PBMC with PHA in combination with bacterial supernatants. The PHA-response was augmented by the supernatant from the proliferative strain M37, while completely abolished by supernatants at 1:50 dilutions from the other strains (Fig. 1a), indicating a cytotoxic effect towards PBMC. This was also reflected in the LDH-cytotoxicity assay, where PUNE08 induced significantly higher cytotoxicity in human PBMC, neutrophils (PMN) and keratinocytes (N/TERT-1 cells) (Fig. 1b). This was in line with increased toxin levels in supernatants from PUNE08 as compared to M37 (Supplementary Table 1). The higher toxin production was also evident as judged by the larger hemolysis zones around PUNE08 colonies on sheep blood agar as compared to M37 (Fig. 1c and Supplementary Fig. 1e). The two reference strains showed hemolysis and cytotoxic patterns resembling that of PUNE08, i.e. larger hemolysis zones and greater cytotoxicity as compared to M37 (Fig. 1a–c).

To identify the genetic factor responsible for this phenotypic diversity the whole genome of PUNE08 and M37 was determined (sequences are deposited in DNA data bank of Japan (DDBJ) and the accession numbers are M37: BCLL01000001-BCLL01000060 and PUNE08: BCLM01000001-BCLM01000056). Sequence analyses revealed a single nucleotide mutation at position 667 of the *agr*C gene in strain M37 compared to PUNE08 leading to an amino acid substitution from tyrosine (Y) to cysteine (C) at position 223 (Fig. 1d). Of note, sequence comparisons of other virulence factors and regulators, including regions of the *agr* loci, were analyzed but did not reveal any additional polymorphism or inactivating mutations*. agr*C gene specific amplification and sequencing of the two European reference strains (HAR22 and NCTC13142) showed that they had the same allele (Y223) as PUNE08 (Fig. 1d). Furthermore, *agr*C sequence analyses of a panel of ST22 MRSA clinical isolates (Supplementary Table 1) revealed that the two proliferative strains, including M37, both had cysteine in position 223 whereas the other strains that were all cytotoxic had tyrosine in this position (Supplementary Fig. 1a).

A molecular model of AgrC protein with Y223C amino acid substitution showed that mutation lies at the dimerization interface of the cytosolic domain of AgrC (Fig. 1e). Solution properties of the purified, recombinant AgrC_Y223C_ protein were examined by size exclusion chromatography and Dynamic Light Scattering. Although the protein was readily purified to homogeneity, it was highly prone to aggregation. This observation is consistent with the size exclusion chromatography profile as well as Dynamic Light Scattering (Supplementary Fig. 1a–d). While the cytosolic domain of the wild-type AgrC is monodispersed, AgrC_Y223C_ is predominantly aggregated in solution. We also note a marked reduction, up to ten fold, in binding affinity of the AgrC_Y223C_ protein with AgrA as compare to the wild-type AgrC (Fig. 1f,g).

### Amino acid substitution in AgrC results in a phenotypic switch

**Figure 2:**
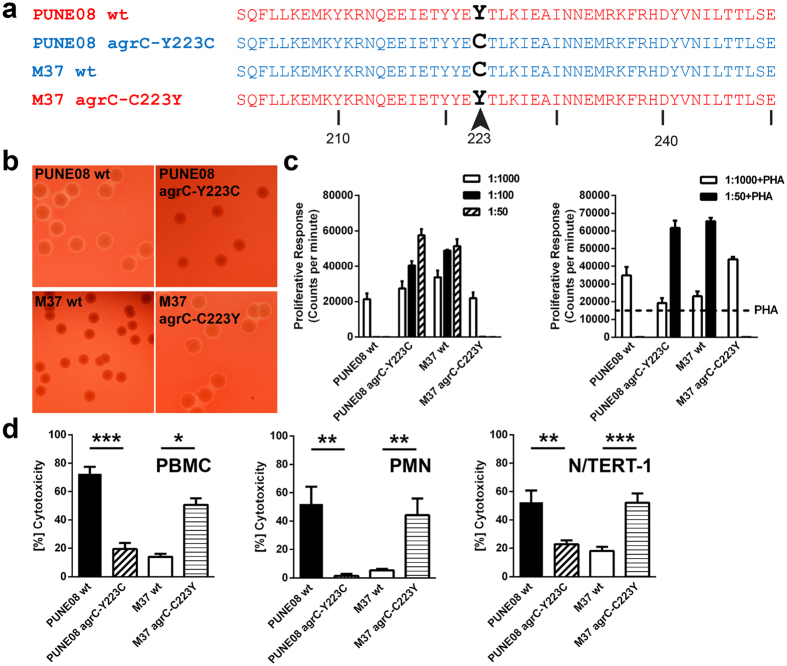
Phenotypic switch of the PUNE08 and M37 *S. aureus* strains due to direct substitution of tyrosine to cysteine or cysteine to tyrosine at the position 223 in AgrC. (**a**) Amino acid sequence analysis of AgrC from indicated wild-type and respective mutant strains. (**b**) Representative images of hemolysis on blood agar plates induced by indicated wild-type and respective mutant *S. aureus* strains (see also Supplementary Fig. 1e). (**c**) Proliferative or cytotoxic responses by human PBMC isolated from three healthy donors stimulated with indicated dilution series of bacterial supernatants. Stimulation without (left panel) and with (right panel) addition of PHA to each supernatant are shown. The data represent the mean values ± s.d. (n  =  3). (**d**) Cytotoxicity induced by bacterial supernatants of 1:50 dilutions towards human PBMC, human neutrophils (PMN) and human keratinocytes (N/TERT-1). Mean values ± s.d. from four or more volunteers (PBMC, PMN) or experiments are shown (n ≥ 4). The statistical significance between wild-type and respective mutant strains was determined using two-tailed Mann-Whitney U-test (*p < 0.05; **p < 0.01; ***p < 0.001).

Through allelic replacement we generated *agrC* mutant strains (Y223C and C223Y) in PUNE08 and M37 genetic backgrounds, respectively (Fig. 2a). The substitution of amino acids was directly associated with toxin production, as assessed by hemolysis on blood agar (Fig. 2b and Supplementary Fig. 1e). In contrast to their respective wild-type strain (wt), the PUNE08 *agr*C-Y223C mutant showed no hemolysis, whereas the M37 *agr*C-C223Y mutant had a clear hemolysis around the colonies. A similar distinct phenotypic switch was evident when culture supernatants from the strains were tested in proliferation and cytotoxicity assays (Fig. 2c,d). M37 wt switched from a proliferative to a cytotoxic profile in the *agr*C mutant, whereas the opposite was noted for PUNE08 wt and its mutant. Taken together, the results demonstrate that the cytotoxic activity is dependent on tyrosine at position 223 of AgrC.

**Figure 3:**
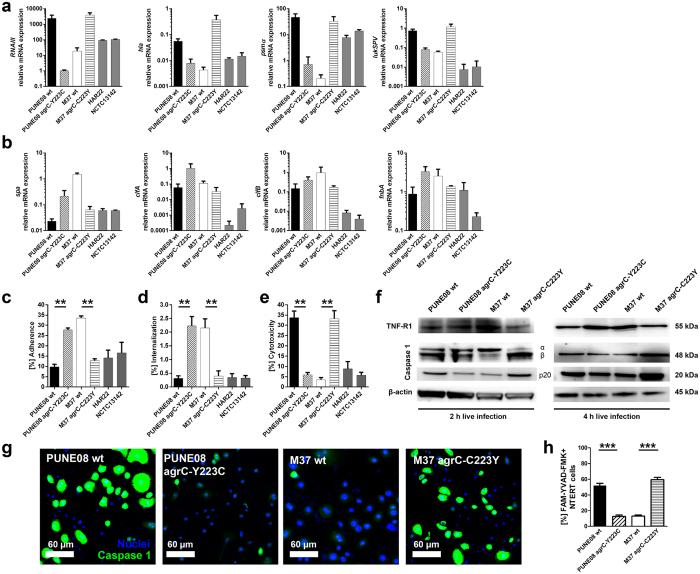
Differential gene expression and virulence profile in strains with different AgrC variants. (**a**) Relative mRNA expression of indicated genes regulating or encoding exotoxins from stationary phase bacterial cultures. The data represent the mean values ± s.d. from three independent experiments (n = 3). (**b**) Relative mRNA expression of indicated genes encoding for surface-attached proteins from stationary phase bacterial cultures. The data represent the mean values ± s.d. from three independent experiments (n = 3). (**c**) Bacterial adherence to, internalization into (**d**) and cytotoxicity towards (**e**) human keratinocytes after two hours of infection. The data represent the mean values ± s.d. (n ≥ 4). The statistical significance between wild-type and respective mutant strains was determined using two-tailed Mann-Whitney U-test (**p < 0.01). (**f**) Representative images of Western Blot analyses of TNF-R1 and Caspase 1 subunits expression after 2 and 4 h of keratinocytes infection. β-actin was used as a loading control (n = 4). Uncropped full-size blots are shown in supplementary Fig. 2. (**g**) Representative images of caspase 1 activation after 2 h of keratinocytes monolayer infection with indicated strains (n = 3). (**h**) Quantitative analysis of caspase 1 positive cells after 2 h of infection. Number of cells were calculated in multiple fields (n = 5). The data represent the mean values ± s.d. from three independent infections (n = 3). The statistical significance between wild-type and respective mutant strains was determined using two-tailed Mann-Whitney U-test (***p < 0.001).

To test the impact of the *agrC* mutation on virulence gene expression, a panel of genes encoding exotoxins and cell surface proteins was analyzed in stationary phase bacterial cultures. Consistent with the noted cytotoxicity, strains harboring the tyrosine at position 223 of AgrC showed a higher abundance of *RNAIII*, as well as exotoxin transcripts e.g. *hla*, *psmα*, and *lukSPV* (Fig. 3a). In contrast, strains with the *agr*C-Y223C variant expressed higher transcript levels of cell surface proteins (e.g. *spa*, *clfA*, *clfB*, and *fnbA*) and lower *RNAIII* and exotoxin transcripts (Fig. 3b); thus, indicating a colonizing phenotype. In line with this assumption, live infections with M37 wt and PUNE08 *agr*C-Y223C showed significantly higher adherence to and internalization into keratinocytes than PUNE08 wt and M37 *agr*C-C223Y (Fig. 3c,d). Similar to previous data using bacterial supernatants, live infection with PUNE08 wt and M37 *agr*C-C223Y triggered significantly higher cytotoxicity (Fig. 3e). As staphylococcal protein A (SPA) has been reported to interact with and upregulate TNF-R1^29^,^30^, and the toxin mediated cytotoxicity has been linked to inflammasome/caspase-1 activation^31^,^32^, these were selected as markers for a differential host response. In agreement with bacterial gene expression data, keratinocytes infected with M37 wt and PUNE08 *agr*C-Y223C (i.e. strains with upregulated *spa*) showed increased amounts of TNF-R1 levels, whereas PUNE08 and M37 *agr*C-C223Y (i.e. strains with upregulated toxin genes) showed augmented pro-caspase 1 (α and β) and active p20 subunit levels (Fig. 3f and Supplementary Fig. 2). In addition, activation of caspase 1 was measured using fluorescent probe carboxyfluorescein (FMK)-YVAD-fluoromethyl ketone (FMK). The percentage of FAM-YVAD-FMK positive cells was significantly higher in PUNE08 wt and M37 *agr*C-C223Y infected cultures (Fig. 3g,h and Supplementary Fig. 3). Based on these findings we postulate that (i) strains harboring tyrosine in position 223 of AgrC are more cytotoxic in nature, whereas (ii) strains harboring the cysteine in this position have characteristics of a colonizing phenotype.

### Cytotoxic strains harboring tyrosine in position 223 of AgrC cause more severe tissue damage in a human 3D skin model

**Figure 4:**
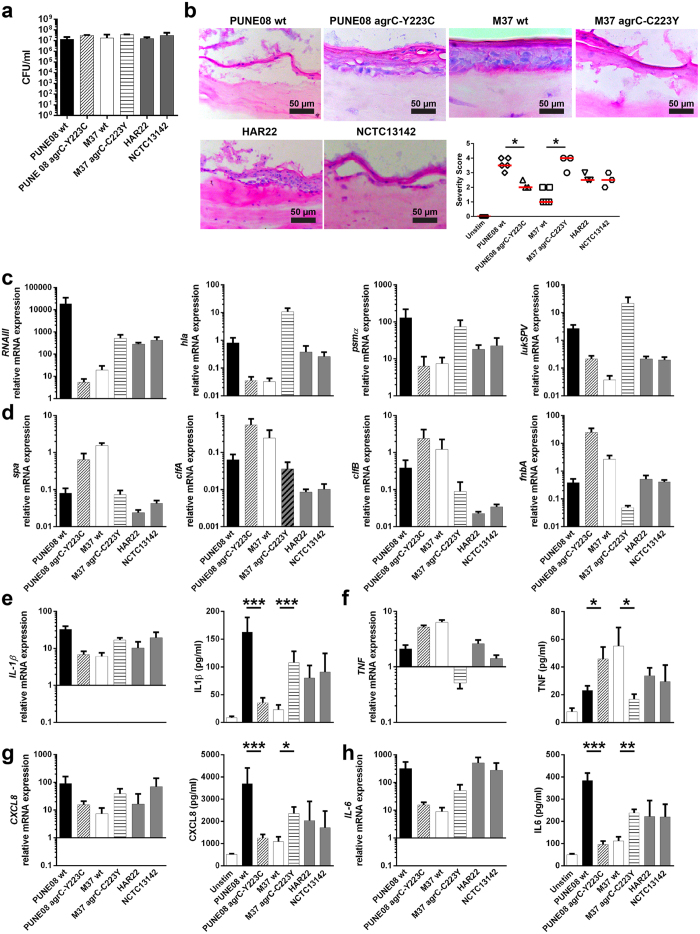
AgrC variation determines the severity of infection in skin tissue model. (**a**) Total CFU counts of bacteria recovered from skin tissue models after 24 h of infection with indicated *S. aureus* strains. The data represent the mean values ± s.d. (n ≥ 3). (**b**) Histological analysis of the skin tissue models after infection. Representative images 24 h post infection and blinded scoring analysis with indicated strains are shown (n ≥ 3). Horizontal lines denote median values. The statistical significance between wild-type and respective mutant strains was determined using two-tailed Mann-Whitney U-test (*p < 0.05). (**c**) Relative mRNA expression of indicated genes regulating or encoding exotoxins during the 24 h skin tissue model infection. The data represent the mean values ± s.d. from three independent infections (n = 3). (**d**) Relative mRNA expression of indicated genes encoding for surface-attached proteins during the 24 h skin tissue model infection. The data represent the mean values ± s.d. from three independent infections (n = 3). (**e**) IL-1β, (**f**) TNF, (**g**) CXCL8, and (**h**) IL-6 relative mRNA expression compared to unstimulated model (black line; left panel) and protein (right panel) released in response to infection from skin tissue model supernatants. The data represent the mean values ± s.d. (n≥3). The statistical significance between wild-type and respective mutant strains was determined using two-tailed Mann-Whitney U-test (*p < 0.05; **p < 0.01; ***p < 0.001).

To test this hypothesis, human organotypic skin tissue with a stratified epithelium and a dermal fibroblast layer was infected with the different strains. All six strains readily infected the skin tissue and equal bacterial load was detected 24 h post infection (Fig. 4a). Histological analyses of hematoxylin/eosin stained tissue revealed that all cytotoxic strains elicited more severe tissue damage characterized by epithelial disruption and detachment as compared to the less cytotoxic strains (Fig. 4b). Gene expression analyses of the infected skin tissue model (Fig. 4c,d) revealed a similar differential virulence gene expression profile as noted in stationary phase bacterial cultures (Fig. 3a,b). In addition, IL-1β, TNF, CXCL8 and IL-6 were assessed at mRNA and protein levels in infected skin models. This revealed that the cytotoxic PUNE08 wt and M37 *agr*C-C223Y strains induced higher IL-1β, CXCL8, and IL-6 as compared to the colonizing M37 wt and PUNE08 *agr*C-Y223C (Fig. 4e–h), whereas the reverse was true for TNF (Fig. 4f). Thus the data are in agreement with the noted activated caspase-1 versus upregulated TNF-R1 expression in infected keratinocytes.

### Colonizing strains harboring cysteine in position 223 of AgrC show a persistence phenotype in a human 3D skin model

**Figure 5:**
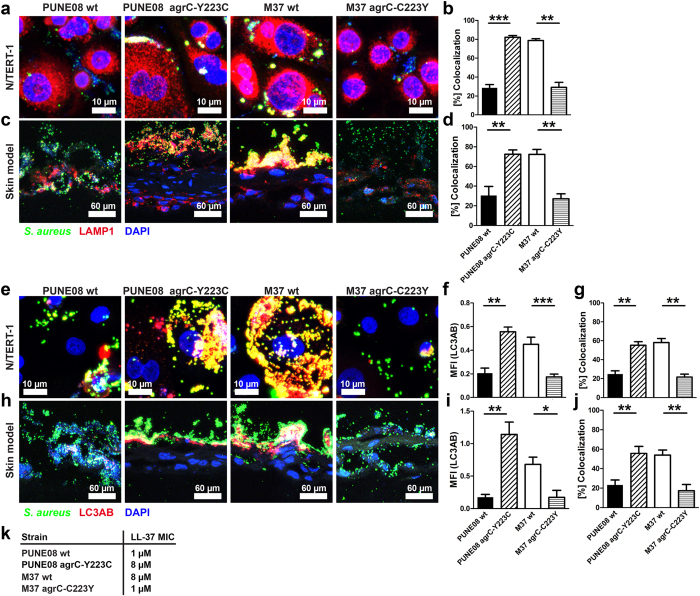
Bacterial intracellular localization and autophagy induction by strains with different AgrC variants. (**a**,**b**) Representative micrographs and quantitative analysis of LAMP1 and *S. aureus* co-localization in infected keratinocytes monolayers after 2 h of infection. (**c**,**d**) Representative micrographs and quantitative analysis of LAMP1 and *S. aureus* co-localization in infected skin tissue models after 24 h of infection. (**e–g**) Representative micrographs, quantitative analysis of MFI of LC3AB, and analysis of LC3AB and *S. aureus* co-localization in infected keratinocytes monolayers after 2 h of infection. (**h–j**) Representative micrographs, quantitative analysis of MFI of LC3AB, and analysis of LC3AB and *S. aureus* co-localization in infected skin tissue models after 24 h of infection. (**k**) The MIC of LL-37 towards the *S. aureus* were determined from two independent experiments performed in triplicates. (**a**–**j**) The data represent the mean values ± s.d. from three independent infections (n = 3). The statistical significance between wild-type and respective mutant strains was determined using two-tailed Mann-Whitney U-test (*p < 0.05; **p < 0.01; ***p < 0.001).

To further investigate the noted difference in adherence and internalization between the strains, imaging studies were performed in infected keratinocytes and organotypic skin tissue. We focused on LAMP-1 and autophagy as both of these have been implicated in intracellular persistence^24^,^33^. Confocal analyses revealed that M37 wt and PUNE08 *agr*C-Y223C strains strongly co-localized with both LAMP1 as well as with the autophagosome marker LC3 (Fig. 5a–j, Supplementary Figs 4–7). In contrast, only minor co-localization with LAMP1 or LC3 was observed in cells or tissue infected with the cytotoxic PUNE08 wt and M37 *agr*C-C223Y strains. In addition, a strong induction of autophagy was seen exclusively for M37 wt and PUNE08 *agr*C-Y223C strains (Fig. 5e–j, Supplementary Figs 6 and 7).

Another common property associated with colonization and persistence is resistance against antibiotics and antimicrobials, such as the LL-37 which is abundantly expressed in skin^34^,^35^,^36^,^37^. Determination of minimum inhibitory concentrations (MIC) of LL-37 revealed that the M37 wt and PUNE08 *agr*C-Y223C strains were less susceptible to LL-37 than PUNE08 wt and M37 *agr*C-C223Y (Fig. 5k). Taken together, the data support an enhanced colonizing/persistent phenotype of M37 wt and PUNE08 *agr*C-Y223C strains, associated with an enhanced exploitation of autophagy and decreased susceptibility to LL-37.

### Phenotypic AgrC variants cause varying disease severity *in vivo*

**Figure 6:**
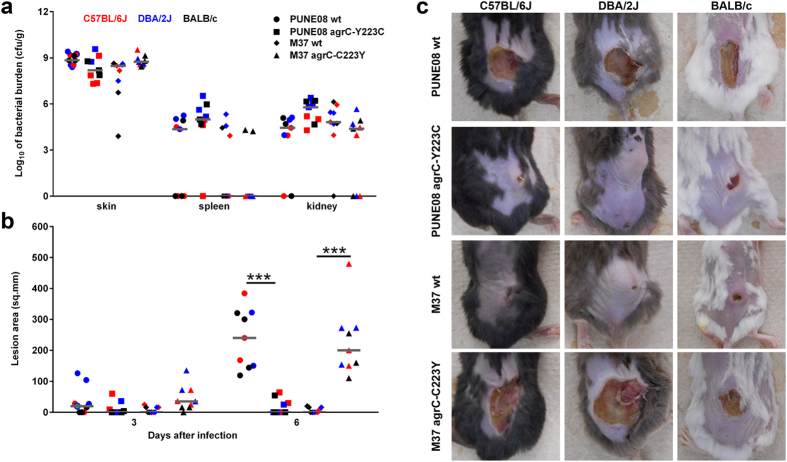
*In vivo* infections with *S. aureus* strains harboring tyrosine at position 223 of AgrC result in severe tissue damage. (**a**) Bacterial loads and dissemination from indicated mice strains infected with indicated *S. aureus* strains. (**b**) Lesion sizes of indicated mice strains infected with indicated *S. aureus* strains at days 3 and 6 post infection. (**c**) Representative images of lesion sizes at day 6 post infection. In total, three mice strains (C57BL/6J [red], DBA/2J [blue], and BALB/c [black]) were used. From each group three mice were infected with indicated *S. aureus* strain and the infection was monitored over a period of six days. Each symbol represents one mouse and horizontal lines represent the median value. The statistical significance between wild-type and respective mutant strains was determined using two-tailed Mann-Whitney U-test (***p < 0.001).

To verify the *in vitro* findings in an *in vivo* skin infection model, three mouse lines of different genetic backgrounds (C57BL/6J, DBA/2J, and BALB/c) were infected subcutaneously with PUNE08 wt, M37 wt, and their respective mutant strains. All mice survived a six days infection period. After euthanization the CFU numbers derived from skin, spleen, and kidney were analyzed. This revealed no significant differences between the bacterial strains in terms of skin colonization or systemic spread (Fig. 6a). However, comparison of lesion sizes six days post infection revealed significantly larger lesions induced by the cytotoxic strains PUNE08 wt and M37 *agr*C-C223Y as compared to M37 wt and PUNE08 *agr*C-Y223C in all mice analyzed (Fig. 6b,c).

## Discussion

In this study we characterize two clinical ST22 MRSA strains associated with skin and soft tissue infections displaying starkly different phenotypic response profiles despite having identical virulence gene profiles. We identify that a single amino acid substitution in the AgrC (tyrosine to cysteine at position 223) leads to a destabilization of the AgrC-AgrA interaction with a subsequent differential regulation of the virulence gene repertoire resulting in a switch from a cytotoxic, toxin-mediated phenotype to a colonizing phenotype. The cytotoxic variant was isolated from a patient with acute soft tissue infection, while the colonizing variant from a patient with repeated recurrent skin infections.

The Agr is a key regulatory system of virulence gene expression in *S. aureus* and both *agr*C and *agrA* regions are hot spots for single point mutations^22^. Phosphorylation of AgrA by AgrC triggers the expression profile in response to a quorum stimulus^38^. Phosphorylated AgrA is a dimer in solution and preferentially induces the expression of phenol soluble modulins (PSMs)^39^ as well as that of RNAIII, the effector RNA that governs the expression of multiple genes. Typically a high RNAIII will result in a reduced expression of cell surface proteins while increased expression of exotoxin genes^39^. Of note, a G55R substitution in AgrC has been observed among most contemporary CC30 isolates^25^, and a L184I substitution has been identified in the derived clade of the community-acquired MRSA ST80 lineage^40^. However the point mutation at position 223 of AgrC identified and characterized in this report has to our knowledge not been reported previously. AgrC with tyrosine at position 223 was the most common variant being identified in 8 out of the 10 ST22 strains analyzed including the two EMRSA-15 reference strains. We demonstrate through structural and biophysical studies that the Y223C substitution affects the oligomeric assembly of AgrC due to aggregation in solution. The poor interaction between the aggregated AgrC_Y223C_ protein and response regulator AgrA is likely to contribute to the noted differential gene expression. Our data demonstrate that tyrosine at position 223 of AgrC plays an important role in high toxin expression profile of ST22 MRSA resulting in increased cytotoxicity against varying cell types and more severe tissue damage both *in vitro* and *in vivo*. In contrast, strains harboring cysteine at position 223 of AgrC have upregulated genes encoding for cell surface proteins resulting in increased adherence and internalization by keratinocytes; characteristics of a colonizing/persistent phenotype^41^,^42^,^43^. One of the upregulated genes was *spa*, and consistent with the known interaction of SPA with TNF-R1, TNF was more abundant at the mRNA and protein level in tissue models infected with strains exhibiting colonizing phenotype.

Furthermore, our data demonstrate that colonizing and cytotoxic strains differed with respect to intracellular locality as the colonizing strains showed a greater co-localization with LAMP1 as well as with LC3-positive autophagic compartments; features usually associated with more persistent strains. There was also a marked induction of autophagy by the colonizing, but not cytotoxic, strains. In addition, reduced caspase 1 and IL1-β levels were noted in cells and tissue infected with strains having a colonizing phenotype, while both were strongly induced following infection with the cytotoxic strains. This variation is consistent with the link between autophagy and consumption of inflammasome components^24^,^44^,^45^. Considering that inflammasome activation has been reported as a mechanism of *S. aureus* clearance^31^,^32^,^46^, our data would imply that this protective host response is less pronounced in infections with strains having a colonizing/persistent phenotype as compared to a cytotoxic phenotype. In addition, the colonizing phenotype was also linked to a decreased susceptibility to the antimicrobial peptide LL-37. This peptide has been ascribed a critical role in the immune defense mechanisms in bacterial infections in the skin^36^,^37^. The exact mechanism of resistance towards LL-37 warrants further studies.

Overall our data from ST22 *S. aureus* strains show that natural AgrC variants with starkly different phenotypes exist among clinical strains. The colinizing/persistent phenotype was identified in a recurrent skin infection case, while the cytotoxic phenotype was recovered from a patient with an acute soft tissue infection. The cytotoxic phenotype elicited severe tissue pathology both in a human skin tissue model as well as in an *in vivo* murine model. The murine model revealed significantly larger tissue lesions with the cytotoxic as compared to the colonizing/persistent strains. In other reports, single point mutations in the *agr* locus or the *hla* gene were found to contribute to attenuated virulence and long term colonization abilities in clinical isolates belonging to other successful lineages such as contemporary CC30 and USA300 (ST8) isolates^24^,^25^. Whether the phenotypic AgrC variation described here represents a mechanism underlying the success of ST22 as a globally disseminating MRSA clone seems plausible but remains to be proven. Whether this phenotypic switch represents a mechanism for which new variants might emerge, such that they provide a reservoir for dissemination and become difficult to eradicate, thereby contributing to the ongoing persistence of ST22 and other successful MRSA clones, warrants further studies.

## Methods

### Ethics statement

Blood samples from healthy volunteers or buffy coats of blood provided by the blood bank at the Karolinska University Hospital were used. The buffy coats were provided anonymously. In case of healthy volunteers, donors were individuals well acquainted with the research conducted; thus, verbal informed consent was deemed sufficient. The ethical research committee at Huddinge University Hospital (Forskningskommitté Syd) approved the study including this consent procedure.

All animal experiments were carried out in accordance with the recommendations in the Guide for the Care and Use of Laboratory Animals of the National Institutes of Health and with the prior approval of the Institutional Animal Care and Use Committee of the University of North Dakota, Grand Forks, ND 58202 (UND approval number 1310-01).

All experiments were carried out in accordance with the approved guidelines.

### Bacterial strains, eukaryotic cells and culture conditions

*S. aureus* clinical isolates used in this study are summarized in Supplementary Table 1. The strains were cultured overnight at 37 in casein hydrolysate and yeast extract (CCY) medium. Cell-free supernatants were prepared through centrifugation at 3350 × g followed by filtration (0.25 μm).

The human keratinocyte cells (N/TERT-1; a gift from Dr. J. Rheinwald and the Cell Culture Core of the Harvard Skin Disease Research Centre, Boston, MA; Immortalized cells from genetically normal individuals; Phenotypical analysis was done by the reference lab mentioned above) were maintained in EpiLife medium (Invitrogen). Normal human dermal fibroblasts (primary cell lines cultured from genetically normal individuals) were cultured in DMEM (Invitrogen) supplemented with 10% (v/v) fetal bovine serum (FBS; Invitrogen). Both were cultured at 37 °C under a 5% CO_2_ atmosphere. Mycoplasma free cells were cultured under antibiotic free conditions. The cells were tested every month (ATCC Universal Mycoplasma detection kit; Cat No: 30-1012K).

Human neutrophils and PBMCs were isolated from peripheral blood collected from healthy donors by Polymorphprep or Ficoll-Hypaque gradient centrifugation (Axis-Shields), respectively. Both cell types were cultured in RPMI 1640 media (HyClone) supplemented with 5% (v/v) FCS.

### Mutagenesis and production of recombinant AgrC proteins

Recombinant wild-type AgrC protein was expressed and purified as previously detailed^38^. The AgrC_Y223C_ protein was obtained by site-directed mutagenesis of the wild-type AgrC construct using the primers Y223C_F(5′TGTACATTGAAGATTGAAGCTATCAACAACGAAATG3′)/Y223C R(5′TTCATAATAGGTTTCAATTTCTTCTTGATTACGTTTATATTTC-3′). The resulting site directed mutated plasmid was verified by sequencing. Recombinant AgrC_Y223C_ protein was expressed in *Escherichia coli* BL21 DE3* cells and was purified by Ni-NTA metal affinity chromatography as detailed^38^.

### Bio-layer Interferometry

The interaction kinetics of AgrC_Y223C_ with AgrA_FL_ (ligand) was analyzed by bio-layer interferometry. AgrA_FL_ was immobilized on a Amine reactive 2^nd^ generation (AR2G) tips (Fortebio, Inc). Two reference tips, one with the ligand and the other with highest concentration of analyte were used. These experiments were performed in a buffer containing 20 mM HEPES, pH 7.6, 200 mM KCl, 2% glycerol. Both AgrC_Y223C_ and AgrC_wt_ were incubated separately with 5 μM of DTT and H_2_O_2_ respectively in these experiments. The binding kinetics were analyzed using the Octet Data Analysis Software v 8.0.

### Whole genome sequencing and analyses

Whole genome sequencing of *S. aureus* M37 and PUNE08 isolates was performed using massively parallel high throughput sequencer Illumina Hiseq-1000. The raw-reads for both isolates were filtered for quality and more than, 8 million reads were obtained with at-least 100X coverage for the 2.8 Mb *S. aureus* genome. The raw reads were assembled into contigs using VELVET version 1.25^47^ and gene predictions were made using GLIMMER 3.02^48^. The protein sequences were derived from the above gene sequences using EMBOSS transeq^49^ the relative arrangement of the contigs was determined by BLASTP and TBLASTN^50^. The sequences are deposited in the DNA data bank of Japan (DDBJ) and the accession numbers are M37 wt: BCLL01000001-BCLL01000060 and PUNE08 wt: BCLM01000001-BCLM01000056.

In total, 14 genes (*spa, hla, hld, hlg, pvl, psmα and β, sarA, rot, sigB, agrA, B, C and D)* associated with virulence or regulation of virulence factor expression were analyzed. After the BLASTP/BLASTN prediction of the genes, 1 kb upstream/downstream region sequences (to include the promoter region also) were extracted from the contig sequences using PERL script. The extracted sequences were aligned using online CLUSTAL W tool.

### Targeted *agr*C sequence analysis

For *agrC* gene specific sequencing, DNA from ST22 *S. aureus* strains was isolated using DNA Blood and Tissue Kit (Qiagen) according to manufacturer’s instructions and the *agr*C gene was (i) amplified by using primers presented in Supplemental Table 3 and (ii) the sequencing was performed by SourceBioscience (Germany). The extracted sequences were aligned using online CLUSTAL W tool.

### Construction of the mutant strains by allelic exchange of *agrC* in *S. aureus* strains

Plasmids were constructed to swap the PUNE08 *agrC*^Y223^ (Ery^R^) and M37 *agrC*^C223^ (Ery^S^) into the opposing backgrounds. From genomic DNA of either PUNE08 or M37, a 1.1 kb amplicon centered on the *agrC* single nucleotide polymorphism were amplified with primers IM406 (5′-**CCTCACTAAAGGGAACAAAAGCTGGGTACC**CTTGATTATTTTTTCATCATAGTAATTTCG3′)/IM407(**CGACTCACTATAGGGCGAATTGGAGCTC**TTTTAAAGTTGATAGACCTAAACCACG). The gel-purified amplicons were recombined into pIMAY-Z^51^ by SLiCE^52^ and transformed into *E. coli* strain IM08B^51^. The inserts from positive clones were verified by Sanger sequencing. The method of Monk *et al*.^51^ was followed for the transformation and allelic exchange with M37 and PUNE08. Putative allele swaps were initially screened for the gain (M37) or loss (PUNE08) of hemolysis on sheep blood agar with subsequent sequencing of the *agrC* gene to validate the introduction of the SNP. This yielded PUNE08 agrC*-*Y223C and M37 agrC*-*C223Y with both maintaining their parental erythromycin resistance/sensitivity profiles.

### Infection and supernatant stimulations of human cells

Adherence to, internalization into, and exit from eukaryotic cells were quantified using the antibiotic protection assay^53^. Briefly, 24-well plates were inoculated with 2 × 10^5^ N/TERT-1 cells/well in EpiLife without antibiotics. The cells were allowed to grow to confluence. For the assay, the cells were washed and infected with *S. aureus* strains at a multiplicity of infection (MOI) of 1:10. Two hours after infection, the viable counts of *S. aureus* (colony-forming units, CFU) released from lysed cells were determined by plating on blood agar. For the assessment of bacterial internalization, 2 h after infection, the cells were washed with PBS and incubated with media supplemented with lysostaphin (20 μg ml^−1^) and gentamicin (120 μg ml^−1^) for an additional 2 h. Subsequently, the cells were washed and lysed, and the CFU counts were determined as described above.

Cells were stimulated with bacterial supernatants (1:50 diluted). Keratinocytes (1.5 × 10^5^) and PBMCs (8 × 10^5^) were stimulated for 24 h and neutrophils (5 × 10^5^) for 2 h after which supernatants were collected. CCY-stimulated and Triton X-lysed cells were used as negative and positive controls, resp. Cytotoxicity were determined by measurement of the LDH activity via CytoTox 96 Non-Radioactive Cytotoxicity Assay Kit (Promega) according to manufacturer’s guidelines.

For assessment of proliferation, PBMCs (2.5 × 10^5^ cells/well) were seeded in 96-well plates and stimulated for 72 h with varying dilutions of bacterial supernatants (1:10–1:1000). The cells were pulsed with 1 μCi of ^3^H-thymidine (GE Healthcare) for additional 6 h of incubation and the uptake of the ^3^H-thymidine was measured.

### SDS-PAGE and Immuno-detection of TNF-R1 and Caspase 1

2 × 10^5^ N/TERT-1 cells were infected at a MOI of 10 for 2 or 4 h and lysed with NBP-40/TritonX buffer. Samples were normalized to equal amounts of protein and boiled in sample buffer (Invitrogen). As molecular mass marker, pre-stained protein standards (Bio-Rad) were used. The samples were separated by 12% SDS-PAGE and transferred to a PVDF membrane. The membranes were blocked with 3% BSA prior to antibody incubations. Antibody incubations were performed according to manufacturer´s guidelines. Antibodies used in this study: TNF-R1 (C25C1), caspase-1 antibody (Cat No: 2225), cleaved caspase-1 (D57A2), β-Actin (D6A8) (all Cell Signaling Technology,) and secondary anti-rabbit IgG horseradish peroxidase linked Fab fragment (GE Healthcare).

### Detection of caspase-1 activity

The active caspase 1 was detected using FAM-YVAD-FMK staining kit (Immunochemistry Technologies). Keratinocytes were infected with *S. aureus* strains at MOI 1:10 for two hours and further staining experiments were performed according to the manufacturer’s protocol. Active caspase 1 was visualized using Nikon A1 confocal microscope (Nikon Instruments).

### 3D organotypic skin-model and infection

The models were generated following a previously published protocol^54^. The skin models were incubated for up to 7 d at 37 °C under a 5% CO_2_ atmosphere, and the culture medium was replaced every second day in the outer chamber. After air exposure the models were infected with 0.25 × 10^6^ bacteria for 24 h.

### Histological analysis of tissue biopsies

For cryosectioning, skin tissue models were treated with 2.0 M sucrose for 1 h before embedding in optimum cutting temperature compound (Sakura Finetek) followed by freezing in liquid nitrogen and stored at −80 °C. 8 μm cryosections were obtained using a MICROM cryostat HM 560 MV (Carl Zeiss) and fixed in 2% freshly prepared formaldehyde in PBS for 15 min at room temperature or in ice-cold acetone for 2 min at −20 °C.

For histological analysis, the sections were stained for 15 sec in Mayer’s haematoxylin and counterstained for 2 min in eosin. Histological severity scoring was performed in a double blinded manner using the following criteria, 0 unaffected tissue, 0.5–1 mild injury with minor epithelial loosening, 1.5–2 moderate injury with some epithelial disruption, 2.5–3 severe injury with continuous epithelial disruption and some detachment, 4 = extensive injury, massive epithelial disruption and detachment. Bacteria were identified via conventional Gram staining.

### Immunostainings and Confocal Microscopy

Infected cells and skin tissue sections were fixed in 2% formaldehyde in PBS. Immunofluorescence staining was performed using following primary antibodies: Rabbit anti-human LAMP1 (abcam; Cat No: ab24170), Mouse anti-*Staphylococcus aureus* (704)(abcam), Rabbit anti-human LC3A/B (D3U4C) (cell signalling technologies). Specific staining was detected by Alexa 488-conjugated donkey anti-mouse IgG, Alexa 546-conjugated donkey anti-rabbit IgG (3.3 μg/ml) and DNA were stained with DAPI (all from Molecular Probes). The staining’s were visualized using a Nikon A1R confocal microscope (Nikon Instruments). Colocalization between *S. aureus* and LC3AB and LAMP, respectively, was analyzed using the image analysis software CellProfiler (version 2.1.1). In short, images were threshold, smoothed and segmented based on the respective channels for *S. aureus* and LC3AB or LAMP. Based on the segmented area, the fraction of *S. aureus* inside the LC3AB or LAMP positive areas (% colocalization) was calculated. The mean fluorescence intensity (MFI) in 10–15 fields/tissue sections was determined using the image analysis software CellProfiler (version 2.1.1).

### RNA Preparation and Quantitative Reverse Transcription PCR Analysis (qRT-PCR)

Bacterial RNA was isolated using FastRNA Blue (MP Biomedicals) and eukaryotic RNA was isolated using RiboPure RNA purification Kit (Ambion) according to manufacturer´s guidelines. cDNA synthesis was performed using the Superscript first-strand synthesis system for RT-PCR (Invitrogen). Primer sets used are summarized in Supplementary Table 3. The real-time PCR amplification was performed with SYBR GreenER Kit (Invitrogen) using an ABI Prism 7500 sequence detection system (Applied Biosystems). The levels of *gyrA* or β-actin transcription were used for normalization.

### Antimicrobial Susceptibility Testing

The minimum inhibitory concentration (MIC) of LL-37 against ST22 *S. aureus* strains was determined using bacteria from the stationary phase. The experiment was performed by adding LL-37 in different concentrations diluted in 0.1% TFA as described previously^55^. The numbers of CFU were analyzed and MIC was defined as the lowest concentration of LL-37 that significantly reduced the bacterial CFU in relation to peptide-free control cultures^56^.

### TNF, IL-1β, IL-6, and CXCL8 ELISAs

The levels of TNF, IL-1β, IL-6, and CXCL8 in supernatants of infected skin tissue models were measured using human TNF, IL-1β, IL-6, and CXCL8 Quantikine ELISA (R&D Systems) according to manufacturer´s guidelines.

### Subcutaneous infection of mice

One day prior to infection the hair was removed from the dorsal side of the mouse with Nair™ hair removal lotion (Church & Dwight Co., Inc). 12 weeks old mice (n = 3) of different genetic background (C57Bl/6J – 7M/7F; DBA/2J – 8M/6F; BALB/C – 7M/7F) were infected subcutaneously with 1 × 10^8^ CFU/100 μl PBS of PUNE08 wt, PUNE08 agrC-Y223C, M37 wt, and M37 agrC-C223Y each (in total 9 mice infected/bacterial strain). Control animals (n = 6, 2 mice/genetic background) were given injections of sterile PBS alone. Animals were randomly assigned to cages so that each group had 9 mice (3 Balb/c, 3 C57B/6J and 3 DBA/2J). Controls had 2 mice of each. No specific randomization method was used for allocation. The investigator was not blinded to group allocation. After infection, each mouse was housed in an individual cage. Animals were observed twice a day for the next six days for mortality, and lesions size. No animals were excluded from analysis. On day 6 lesion size was measured, photographed and the mice sacrificed using approved methods.

### Statistical analysis

No statistical methods were used to predetermine sample size. Data are presented as mean ± s.d. for the indicated number of experiments. The data were analyzed with use of the Graphpad Prism v.5 software (GraphPad, San Diego, CA). Due to small sample size no normality test was performed and non-parametric two-tailed Mann-Whitney U-test was applied for all comparisons between PUNE08 and M37 or between wild-type strains and their respective mutant, *p* values less than 0.05 were considered significant.

## Additional Information

**How to cite this article**: Mairpady Shambat, S *et al*. A point mutation in AgrC determines cytotoxic or colonizing properties associated with phenotypic variants of ST22 MRSA strains. *Sci. Rep.* **6**, 31360; doi: 10.1038/srep31360 (2016).

## Supplementary Material

Supplementary Information
