# Supplementary material for: A point mutation in AgrC determines cytotoxic or colonizing properties associated with phenotypic variants of ST22 MRSA strains
Source: Sci Rep. 2016 Aug 11;6:31360. doi: 10.1038/srep31360 (PMC4980769; doi:10.1038/srep31360)
Supplement: Supplementary Information [file srep31360-s1.pdf]

Supplementary Information

A point mutation in AgrC determines cytotoxic or colonizing properties associated with phenotypic variants of ST22 MRSA strains.

Srikanth Mairpady Shambat[1,¶], Nikolai Siemens[1,¶], Ian R. Monk[2], Disha B. Mohan[3] , Santhosh Mukundan[4], Karthickeyan Chella Krishnan[4], Sushma Prabhakara[5], Johanna Snäll[1], Angela Kearns[6], Francois Vandenesch[7], Mattias Svensson[1], Malak Kotb[4], Balasubramanian Gopal[3], Gayathri Arakere[5], and Anna Norrby-Teglund[1*]

[¶]These authors contributed equally to this work

[1]Karolinska Institutet, Center for Infectious Medicine, Karolinska University Hospital Huddinge, 141 86, Sweden; [2]Department of Microbiology and Immunology, University of Melbourne, Victoria, 3010, Australia; [3]Molecular Biophysics Unit, Indian Institute of Science, Bangalore, 560012, India; [4]Department of Basic Sciences, School of Medicine and Health Sciences, University of North Dakota, ND, 58202-9037, USA; [5]Society for Innovation and Development, Indian Institute of Science, Bangalore, 560012, India; [6]National Infection Service, Public Health England (PHE), Colindale, London, NW95EQ, UK; [7]CIRI, International Center for Infectiology Research; Inserm, U1111; CNRS, Université Lyon 1; École Normale Supérieure de Lyon; French National Reference Center for Staphylococci, Hospices Civils de Lyon, Lyon, UMR5308, France

* Corresponding author: Anna Norrby-Teglund, Karolinska Institutet, Center for Infectious Medicine, Karolinska University Hospital Huddinge, 141 86, Sweden; phone: +46858583297; Email: Anna.Norrby-Teglund@ki.se

Supplementary Methods

Gel Filtration Chromatography for $AgrC_{Y223C}$

Analytical size exclusion experiments were conducted using Superdex S200 column to determine the oligomeric status of $AgrC_{Y223C}$. Also, $AgrC_{Y223C}$ was incubated with DTT to examine their effect on the Cysteine 223 and therefore on the oligomeric status of $AgrC_{Y223C}$.

Toxin ELISAs

Levels of α-toxin and PVL were determined by ELISA using toxin-specific antibodies provided by GSK Vaccines (Belgium) and bioMérieux R&D Immunodiagnostic (France), respectively[1,2].

SupplementaryTable 1. Strains and plasmids used in this study

Strain/plasmid	Clinical diagnosis	MRSA/MSSA	Response Profile*	α-toxin (μg/ml)#	PVL (μg/ml)#	Reference/Source
**(A) *S. aureus* strains**						
PUNE08 (Sa08)	SSTI	MRSA-IV	C	75	19.6	[3]
PUNE08 agrC-Y223C	-	MRSA-IV	P	N.D	N.D	This study
M37 (Sa37)	SSTI	MRSA-IV	P	9	1.5	[3]
M37 agrC-C223Y	-	MRSA-IV	C	N.D	N.D	This study
Sa6454	SSTI	MRSA-IV	C	18	1.6	This study
SaN08	Meningitis	MRSA-IV	C	32	8	[3]
Sa113	Brain abscess	MRSA-IV	C	20	3.2	[3]
Sa114	Cerebral abscess	MRSA-IV	C	23	4	[3]
Sa754	Invasive infection	MRSA-IV	C	20	1	[3]
Sa165	colonizing strain	MRSA-IV	C	19.3	2.3	[3]
Sa115	colonizing strain	MRSA-IV	P	0.2	0.08	[3]
**(B) EMRSA-15 reference *S. aureus* strains**						
HAR22	SSTI	MRSA-IV	C	N.D	N.D	Provided by Dr. H. Lencastre[4]
NCTC 13142	SSTI	MRSA-IV	C	N.D	N.D	Provided by A Kearns[5]
**(C) *E. coli* strains**						
BL21 (DE3)	-	-				NEB
IM08B	-	-				[6]
**(D) Plasmids**						
pIMAY-Z	-	-				[6]
pET22b						Novagen Inc.
pET28a						[7]

MRSA, methicillin-resistant *S. aureus*; MSSA, methicillin-susceptible *S. aureus*; P, proliferative; C, cytotoxic; SSTI, skin and soft tissue infection. N.D, Not determined; Names in parenthesis for strains are nomenclature based on previous publication

***** The response profile is determined by the pattern of proliferative responses elicited by different dilutions of bacterial supernatants against PBMC. A proliferative profile was denoted if all dilutions elicited a proliferative response. A cytotoxic pattern was denoted if a) proliferation was only noted at the highest dilution of the supernatants, and b) the supernatants (1:50 dilution) resulted in inhibition of PHA-induced responses in co-stimulation experiments.

[#]Amounts of toxins present in supernatants cultures grown to stationary phase (17hours)

SupplementaryTable 2. Toxin gene profile of the clinical ST22 *S. aureus* strains.

Strains	ST/SPA type	*agr* type	*se/tsst-1 /egc*	*pvl*	*luk*D/E	Staphylokinase	Hemolysins	Proteases	SSL genes	MSCRAMM/ Adhesin	Capsule/ Biofilm
PUNE08	ST22/t852	I	-/-/+	+	-	+	*hla,hlb,hld,*	*aur, sspA,B,P*	*ssl 5,7,9*	*clf A,B, cna, eno, fnbA, sdrC,*	5/ *ica A,C,D*
M37	ST22/t852	I	-/-/+	+	-	+	*hla,hlb,hld,*	*aur, sspA,B,P*	*ssl 5,7,9*	*clf A,B, cna, eno, fnbA, sdrC*	5/ *ica A,C,D*

Based on gene microarray data: *se,* staphylococcal enterotoxin; *pvl*, Panton-Valentine leucocidin; *hla*, alpha-hemolysin; *hlb*, beta-hemolysin; *hld*, delta-hemolysin; *spl*, serine protease like; *sspA*, serine protease; *sspB*, cysteine protease staphopain B; *sspP*, staphopain A precursor; *ssl*, staphylococcal superantigen-like; *clf*, clumping factor; *cna*, collagen adhesins; *eno*, enolase; *fib*, fibrinogen binding-protein; *fnbA*, fibronectin A; *sdrC,* serine-aspartate repeat-containing protein C

Supplementary Table 3. qRT-PCR and *agr*-sequencing primers used in this study

Gene	Primer	Sequence (5´-3´)
agrC	AgrC_F	TTATGACTTCGCAGATTATTCTGTACT
	AgrC_R	TAGAAAAACTTTCTTGGAACAATTCAT
hla	hla_F	CGGCACATTTGCACCAATAAGGC
	hla_R	GGTTTAGCCTGGCCTTCAGC
RNAIII	RNAIII_F	GTGATGGAAAATAGTTGATGAGTTGTTT
	RNAIII_R	GAATTTGTTCACTGTGTCGATAATCC
psmα	psmα_F	TATCAAAAGCTTAATCGAACAATTC
	psmα_R	CCCCTTCAAATAAGATGTTCATATC
lukSPV	lukSPV_F	GTGTTGTTCTTCTAGTAGCATGAGT
	lukSPV_R	ATCACATCATTAGGTAAAATGTCTG
spa	spa_F	CAACGGAGTACATGTCGTTA
	spa_R	GCTTGAGCTTTGTTAGCATC
clfA	clfA_F	CAAAGGTGATTTAGCTTTACGTTCAACTT
	clfA_R	TGCTACTTCGTTGTCCCATGAC
clfB	clfB_F	AATGTGTTACCACTTTGATTAGGGTCAA
	clfB_R	GCTGCTGATGCTAAAGGTACAAATG
fnbA	fnbA_F	AACCATTATCCCAAGTTAAGGTATATCCTCT
	fnbA_R	TGGTACTGATGAAGTTGATTTTAGAACACA
IL-6	hIL-6_F	AATTCGGTACATCCTCGACGG
	hIL-6_R	GGTTGTTTTCTGCCAGTGCCT
TNF	hTNF_F	TGGCCCAGGCAGTCAGA
	hTNF_R	GGTTTGCTACAACATGGGCTACA
IL-1β	hIL-1β_F	GCCCTAAACAGATGAAGTGCTC
	hIL-1β_R	GAACCAGCATCTTCCTCAG
CXCL8	hIL-8_F	GAGCACTCCATAAGGCACAAA
	hIL-8_R	ATGGTTCCTTCCGGTGGT
LL37	hLL37-F	TCGGATGCTAACCTCTACCG
	hLL37-R	GTCTGGGTCCCCATCCAT
beta-Actin	hbetaAct_F	CTCTTCCAGCCTTCCTTCCT
	hbetaAct_R	AGCACTGTGTTGGCGTACAG

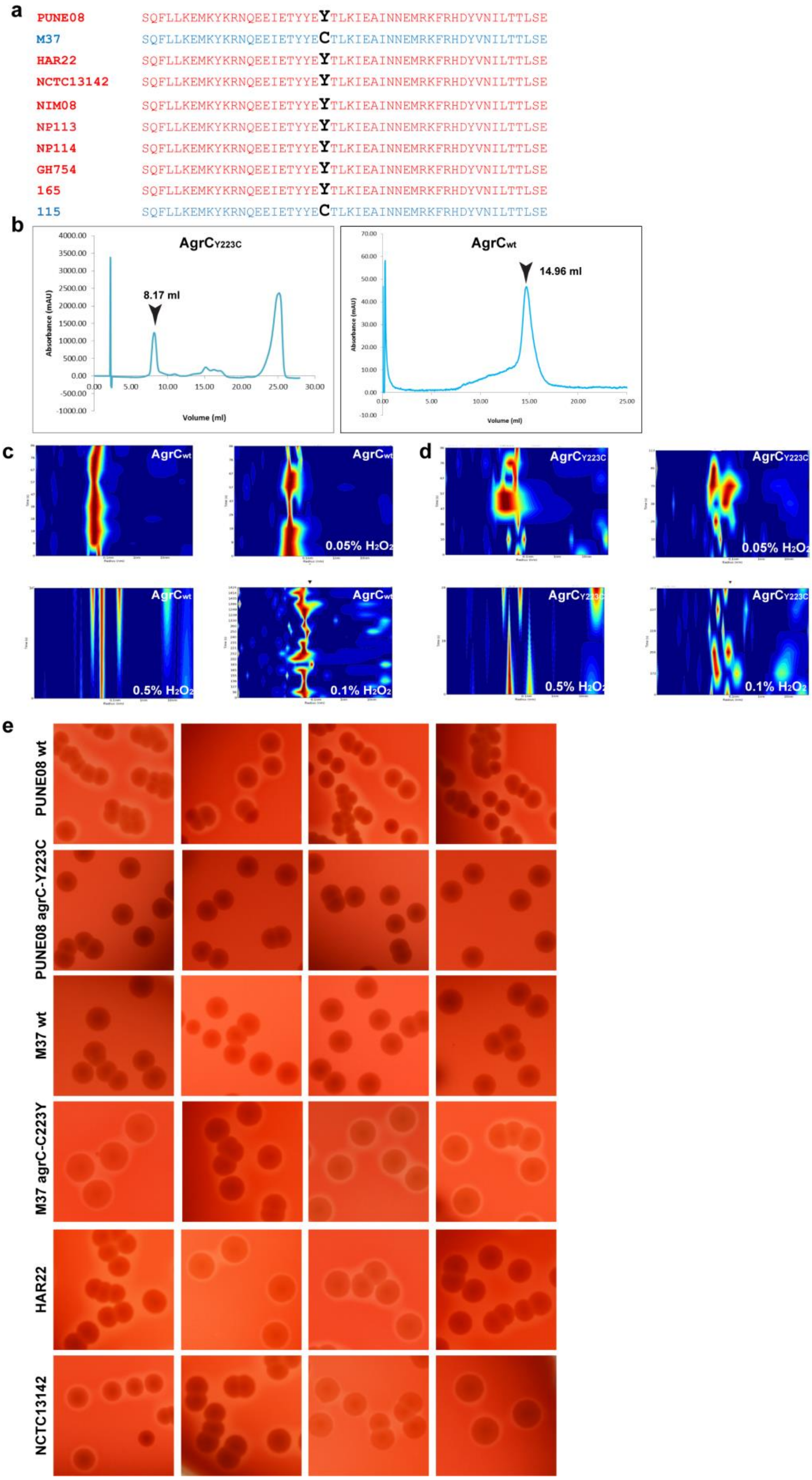
a
PUNE08 SQFLLKEMKYKRNQEEIETYYEYTLKIEAINNEMRKFRHDYVNILTTLSE
M37 SQFLLKEMKYKRNQEEIETYYECTLKIEAINNEMRKFRHDYVNILTTLSE
HAR22 SQFLLKEMKYKRNQEEIETYYEYTLKIEAINNEMRKFRHDYVNILTTLSE
NCTC13142 SQFLLKEMKYKRNQEEIETYYEYTLKIEAINNEMRKFRHDYVNILTTLSE
NIM08 SQFLLKEMKYKRNQEEIETYYEYTLKIEAINNEMRKFRHDYVNILTTLSE
NP113 SQFLLKEMKYKRNQEEIETYYEYTLKIEAINNEMRKFRHDYVNILTTLSE
NP114 SQFLLKEMKYKRNQEEIETYYEYTLKIEAINNEMRKFRHDYVNILTTLSE
GH754 SQFLLKEMKYKRNQEEIETYYEYTLKIEAINNEMRKFRHDYVNILTTLSE
165 SQFLLKEMKYKRNQEEIETYYEYTLKIEAINNEMRKFRHDYVNILTTLSE
115 SQFLLKEMKYKRNQEEIETYYECTLKIEAINNEMRKFRHDYVNILTTLSE
b
AgrCY223C
8.17 ml
Absorbance (mAU)
Volume (ml)
AgrCwt
14.96 ml
Absorbance (mAU)
Volume (ml)
c
AgrCwt
AgrCwt 0.05% H2O2
AgrCwt 0.5% H2O2
AgrCwt 0.1% H2O2
d
AgrCY223C
AgrCY223C 0.05% H2O2
AgrCY223C 0.5% H2O2
AgrCY223C 0.1% H2O2
e
PUNE08 wt
PUNE08 agrC-Y223C
M37 wt
M37 agrC-C223Y
HAR22
NCTC13142

Supplementary Figure 1. Amino acid sequence variation in AgrC results in differential phenotypic response profiles of clinical ST22 *S. aureus* strains. (**a**) Amino acid sequence analysis of AgrC from indicated strains at specified positions. (**b**) Size exclusion profile of $AgrC_{Y223C}$ and wild-type AgrC. The chromatograms reveal that the $AgrC_{Y223C}$ protein is prone to aggregation and elutes in the void volume of the experiment. (**c**) DLS profile for wild-type $AgrC_{t}$ with increasing H_2O_2. (**d**) DLS profile for $AgrC_{Y223C}$ with increasing H_2O_2 concentration. Addition of H_2O_2 seemed to drastically increase the aggregation of $AgrC_{Y223C}$ protein. (**e**) Representative images of hemolysis on blood agar plates induced by indicated clinical and respective mutant *S. aureus* strains.

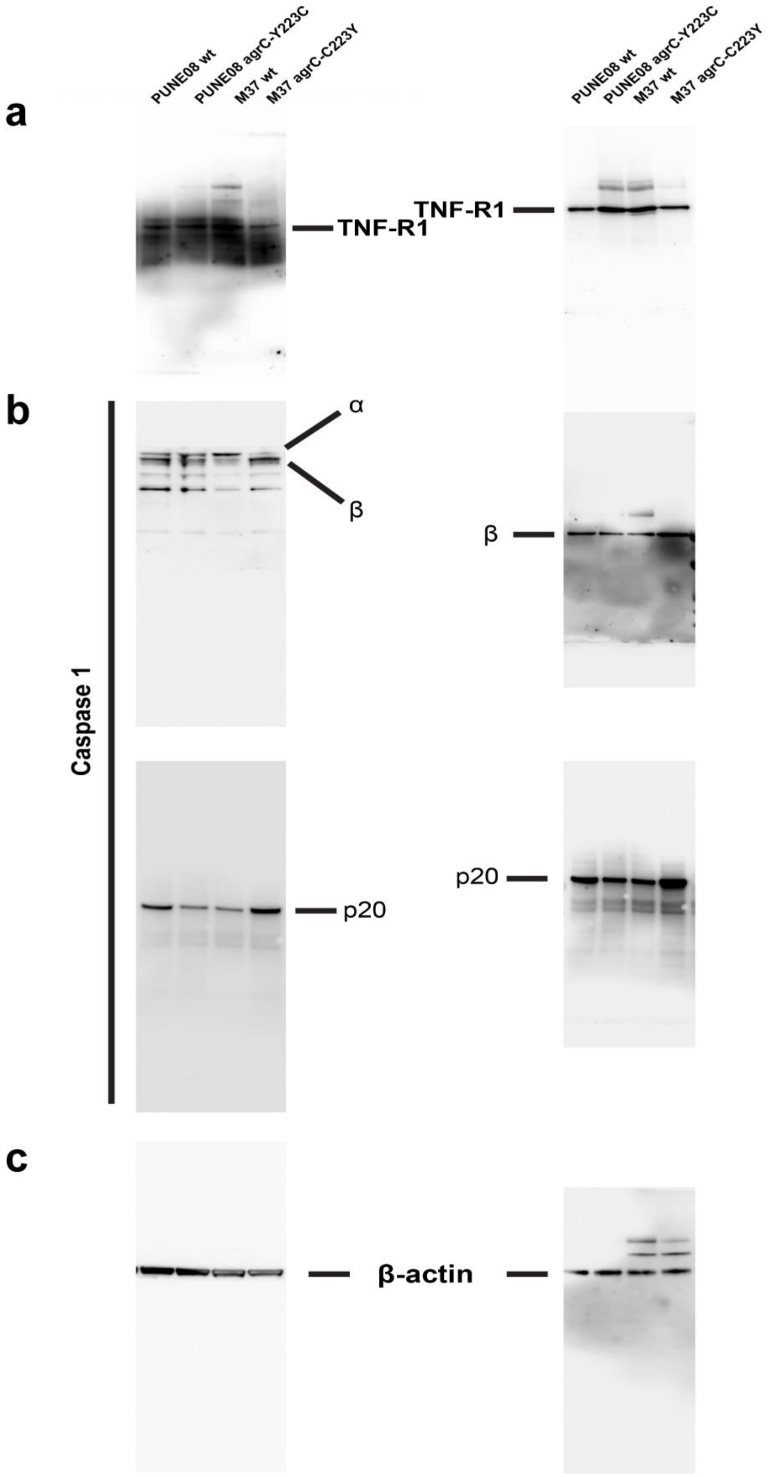

Supplementary Figure 2. Representative images of Western Blot analyses of (**a**) TNF-R1 and (**b**) Caspase 1 subunits expression after 2 h (left panel) and 4 h (right panel) of keratinocytes infection. (**c**) β-Actin was used as a loading control (n=4). Uncropped full-size blots are shown.

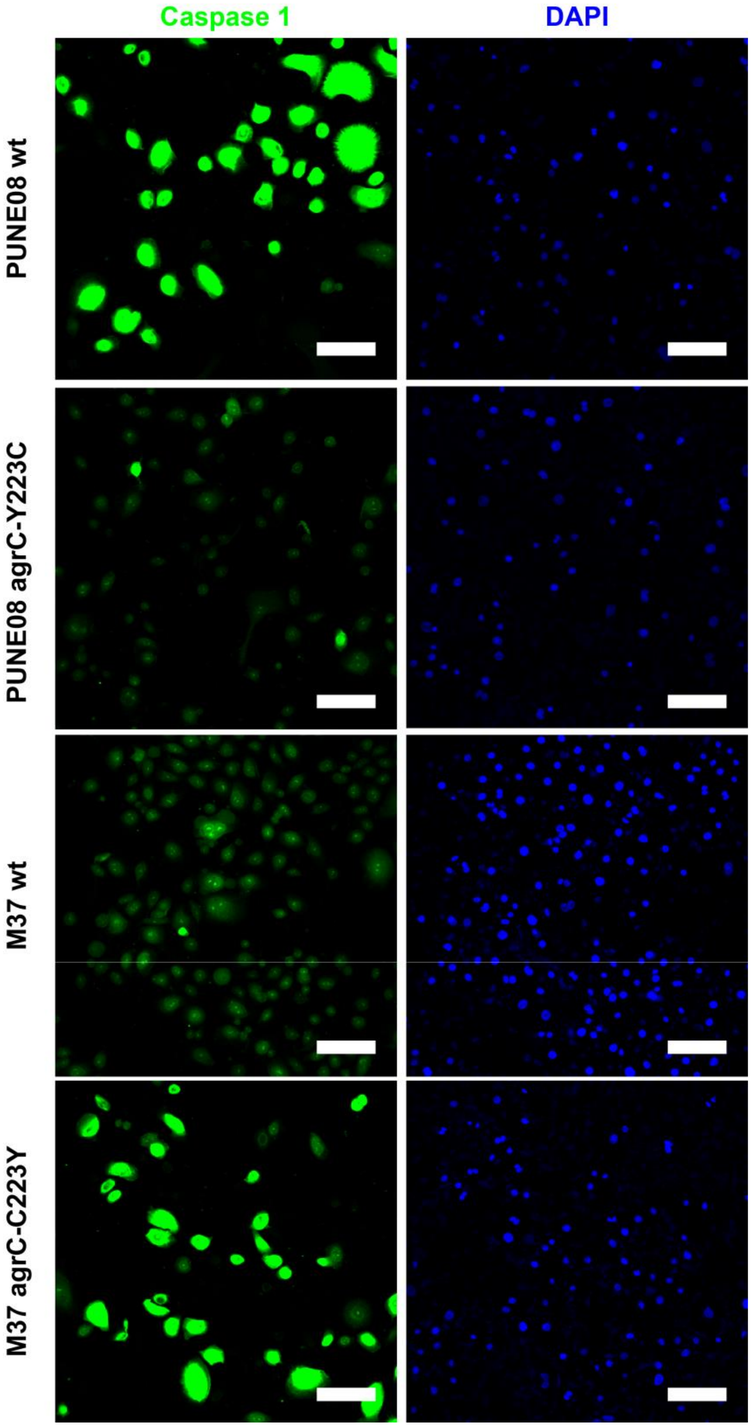

Supplementary Figure 3. Cytotoxic strains induce caspase 1 activation in infected keratinocytes. Representative single channel images of Caspase 1 (green; left panel) secretion and DAPI (blue; right panel) after 2 h of keratinocytes monolayer infection with indicated strains. The scale bars in images equal 60 µm.

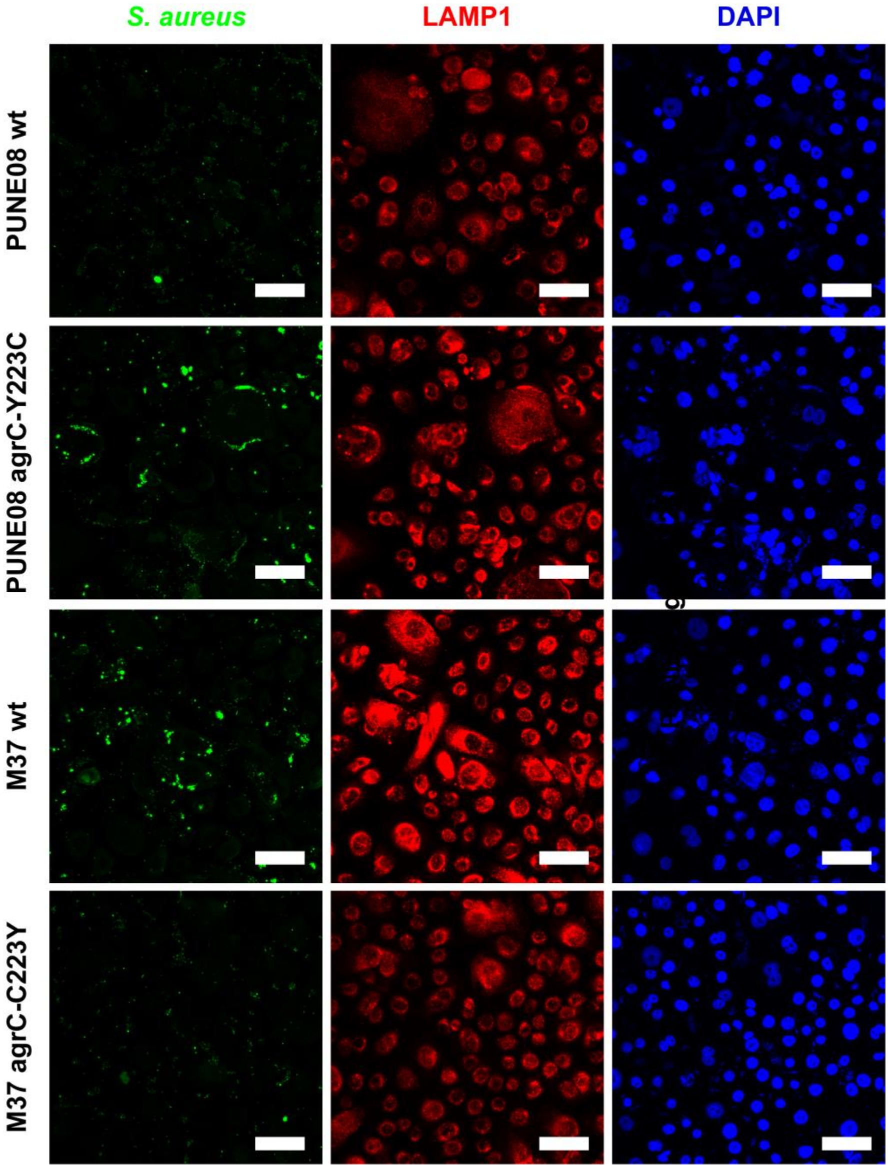

Supplementary Figure 4. *S. aureus* strains exhibiting colonizing phenotype reside within phagolysosome in infected Keratinocytes. Representative single channel images of LAPM1 (red; center panel), DAPI (blue; right panel) and *S. aureus* (green; left panel) co-localization in infected keratinocytes monolayers after 2 h of infection. The scale bars in images equal 60 µm.

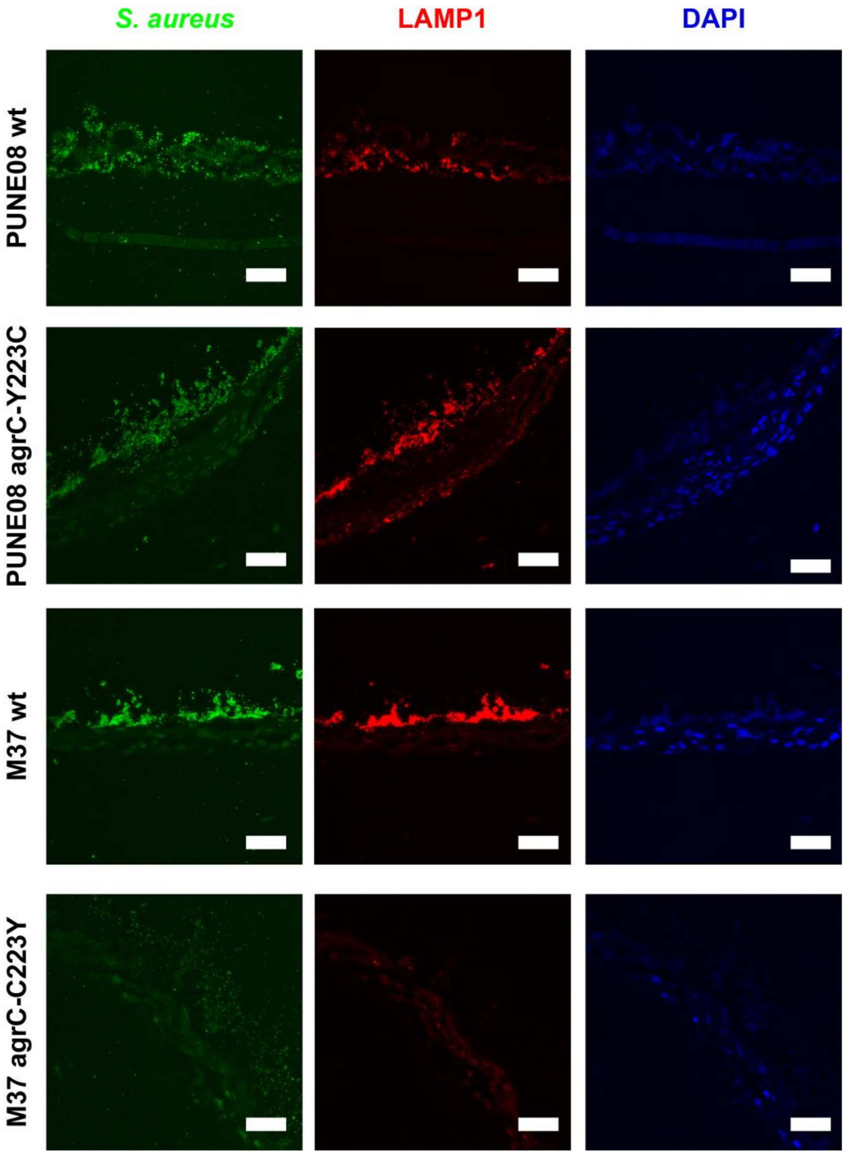

Supplementary Figure 5. *S. aureus* strains exhibiting colonizing phenotype reside within phagolysosome in infected skin tissue models. Representative single channel images of LAPM1 (red; center panel), DAPI (blue; right panel) and *S. aureus* (green; left panel) co-localization in infected skin tissue models after 24 h of infection. The scale bars in images equal 60 µm.

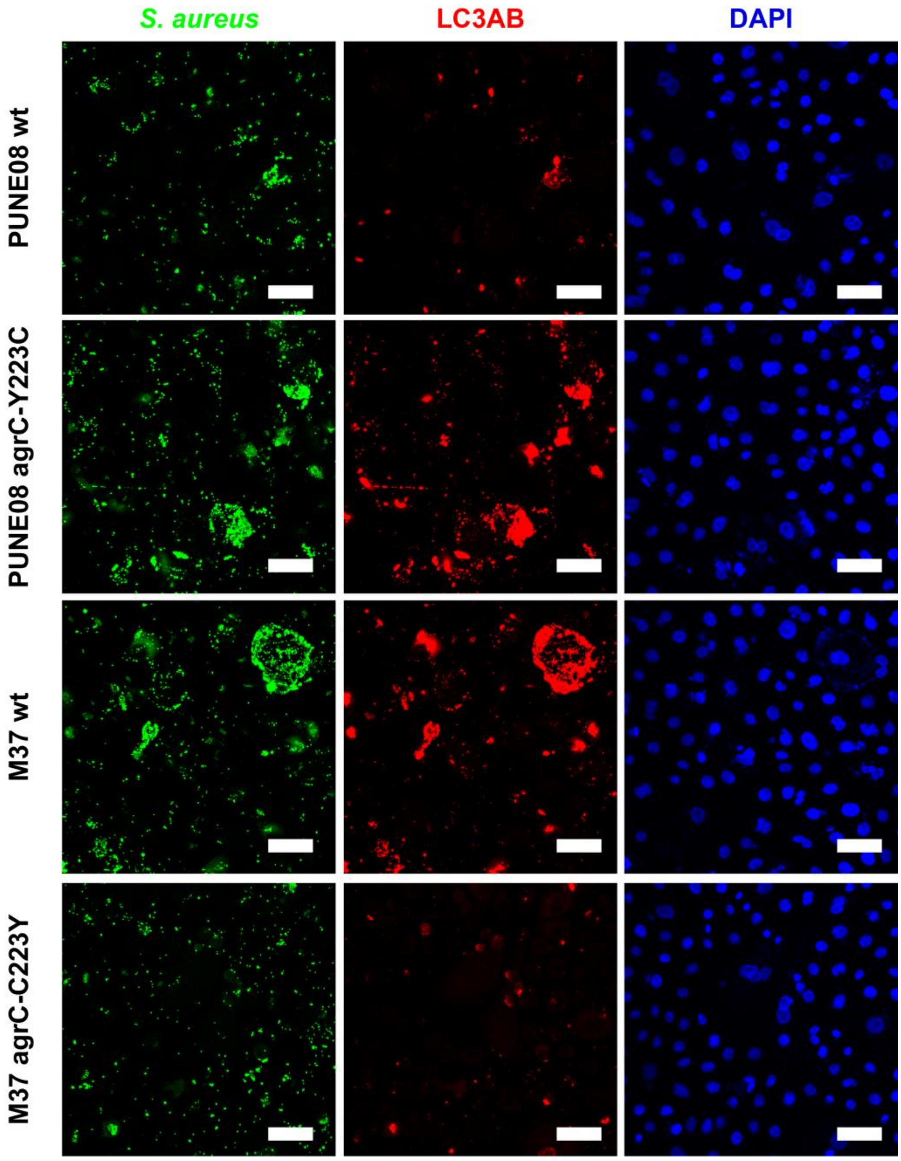

Supplementary Figure 6. *S. aureus* strains exhibiting colonizing phenotype induce autophagy in infected Keratinocytes. Representative single channel images of LC3AB (red; center panel), DAPI (blue; right panel) and *S. aureus* (green; left panel) co-localization in infected keratinocytes monolayers after 2 h of infection. The scale bars in images equal 60 µm.

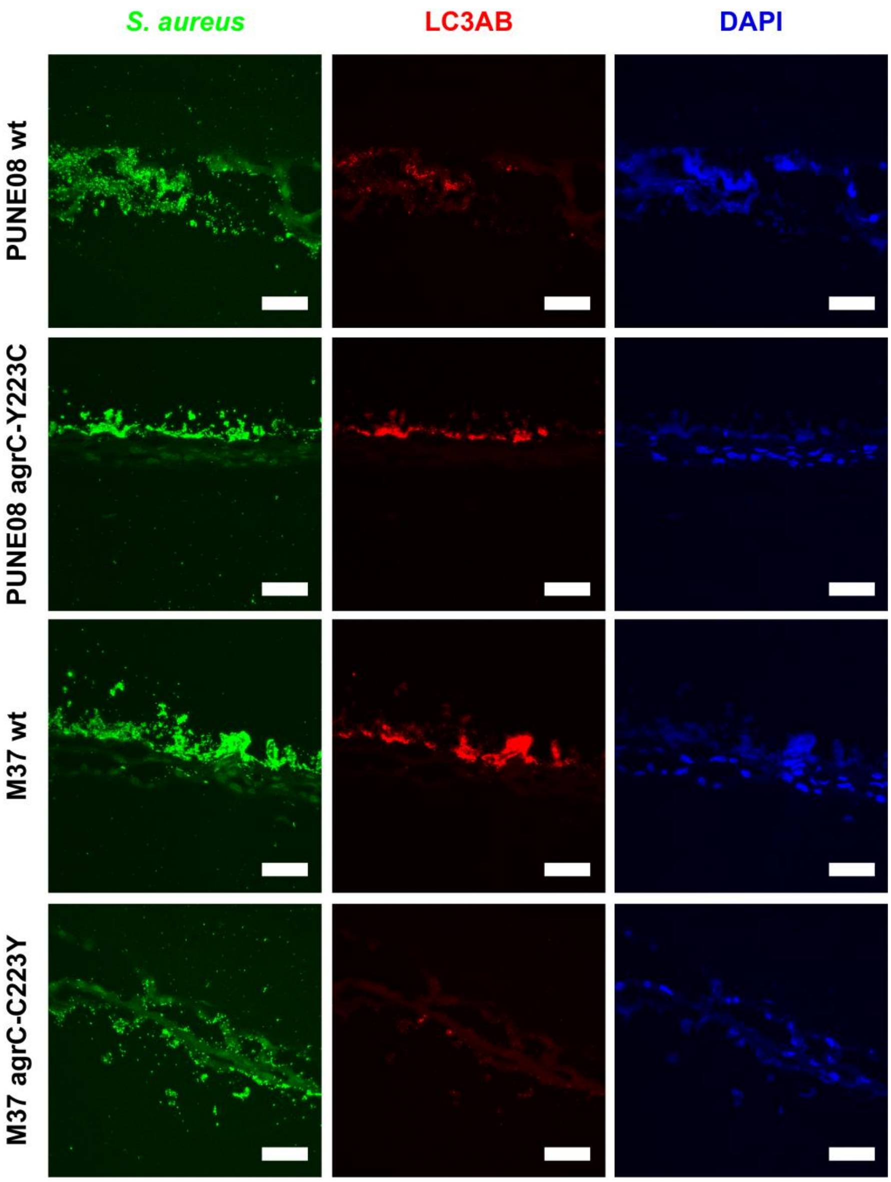

Supplementary Figure 7. *S. aureus* strains exhibiting colonizing phenotype induce autophagy in infected skin tissue models. Representative single channel images of LC3AB (red; center panel), DAPI (blue; right panel) and *S. aureus* (green; left panel) co-localization in infected skin tissue models after 24 h of infection. The scale bars in images equal 60 µm.

References

1. Badiou, C. *et al.* Rapid detection of Staphylococcus aureus Panton-Valentine leukocidin in clinical specimens by enzyme-linked immunosorbent assay and immunochromatographic tests. *J. Clin. Microbiol.* **48**, 1384-1390, doi:10.1128/JCM.02274-09 (2010).
2. Diep, B. A. *et al.* Effects of linezolid on suppressing in vivo production of staphylococcal toxins and improving survival outcomes in a rabbit model of methicillin-resistant Staphylococcus aureus necrotizing pneumonia. *J. Infect. Dis.* **208**, 75-82, doi:10.1093/infdis/jit129 (2013).
3. Mairpady Shambat, S. *et al.* Levels of alpha-toxin correlate with distinct phenotypic response profiles of blood mononuclear cells and with agr background of community-associated Staphylococcus aureus isolates. *PLoS One* **9**, e106107, doi:10.1371/journal.pone.0106107 (2014).
4. Milheirico, C., Oliveira, D. C. & de Lencastre, H. Multiplex PCR strategy for subtyping the staphylococcal cassette chromosome mec type IV in methicillin-resistant Staphylococcus aureus: 'SCCmec IV multiplex'. *J. Antimicrob. Chemother.* **60**, 42-48, doi:10.1093/jac/dkm112 (2007).
5. Jenkins, R., Burton, N. & Cooper, R. Manuka honey inhibits cell division in methicillin-resistant Staphylococcus aureus. *J. Antimicrob. Chemother.* **66**, 2536-2542, doi:10.1093/jac/dkr340 (2011).
6. Monk, I. R., Tree, J. J., Howden, B. P., Stinear, T. P. & Foster, T. J. Complete Bypass of Restriction Systems for Major Staphylococcus aureus Lineages. *MBio* **6**, e00308-00315, doi:10.1128/mBio.00308-15 (2015).
7. Srivastava, S. K., Rajasree, K., Fasim, A., Arakere, G. & Gopal, B. Influence of the AgrC-AgrA complex on the response time of Staphylococcus aureus quorum sensing. *J. Bacteriol.* **196**, 2876-2888, doi:10.1128/JB.01530-14 (2014).
